# Supplementary material for: Patterns of Intron Gain and Loss in Fungi
Source: PLoS Biol. 2004 Nov 30;2(12):e422. doi: 10.1371/journal.pbio.0020422 (PMC532390; doi:10.1371/journal.pbio.0020422)
Supplement: Table S1 — Also available at http://genes.mit.edu/NielsenEtAl/. (4.3 MB ZIP). [file pbio.0020422.st001.zip › NielsenEtAl/html/790.html]

AN6248.1.NCU01516.1.MG03214.1.FG00305.1


```
 CLUSTAL W (1.82) Multiple Sequence Alignments - Introns Inserted


Sequence 1: NCU01516.1	238 aa
Sequence 2: MG03214.1	252 aa
Sequence 3: FG00305.1	244 aa
Sequence 4: AN6248.1	252 aa
Alignment Length: 262 aa
Number Identitical Residues: 83 aa
Alignment Score (without introns) 4298


MG03214.1 	MLRQAVATPSRIARSALRAAGQQRIPSSTPSFRQLPLSAVSRPVRTTSVRWYSAEPESKD
NCU01516.1	MLRTALTRSSRALCSGARVAAQRPIAS---QIFQMQAARTAAPQLRSAARWYSAEAEGEK
FG00305.1 	MFRQAIATSSRALRAAPKVAVPRPFVQ---SQFQAAPAFTLRSAQPAVSRWYSDAKESES
AN6248.1  	MFKRTLLRQAQASRALFSVRSASTAPLAIRQPSQFQPQLLRPFARIPSARCYSTENKTET
          	*:: ::   ::   :   .        :  .  *            .  * **   : : 

MG03214.1 	AAKDAAK---DGEAAAADASPEVALKKELDTKTKEALEWK0DRYLRSVADFRNLQDRQAR
NCU01516.1	KADEGAE---QKEGETDEVA---ALKKQLEAKDAEAREWK0DKCLRTVADFRNLQERTAR
FG00305.1 	KPVEEAKSETKSDEKPAENDPLAELKKSLEAKETEVKDWK0DKCLRTVADFRNLQERTTR
AN6248.1  	QTENGEK-EQKDNGNESTGTADEQCQKELEEKKKEVIELK0DKYVRSVADFLNLQERTKR
          	 . :  : . . :       .    :*.*: *  *. : * *: :*:**** ***:*  *

MG03214.1 	EMKSTRDFAIQKFARDLVDSVDNLERALAMVPADKIKAASDAAKDSETKPEFLQDLVNLY
NCU01516.1	DVKQAKDFAIQKFAKDLVESVDNFERALSVVPQDKLK--------SEEQSEHLKDLVNLY
FG00305.1 	EVKSAKDFAIQKFAKDLVDSVDNLDRALGMVPQEKLK--------VKDRPEGIEDLANLY
AN6248.1  	DMENARNFAIQRFAVDLLESIDNFDRALLAVPKEKLN---------APKTEENKDLLDLV
          	:::.:::****:** **::*:**::***  ** :*::           :.*  :** :* 

MG03214.1 	EGLKMTENILVSTVAKHGLERFNPN--GE-----KFNPNEHEATFMTPQPDKDDNTVFFT
NCU01516.1	EGLKMTESILLSTLKKHGLERIEPE--GE-----VFNPNEHEATFMAPMPDKEHNVVFHV
FG00305.1 	EGLKMTEDILMNTLKKHGLERLSPE--GE-----KFNPNEQEATFMTPQPDKEDGTVFFV
AN6248.1  	DGLKMTQNILLNTLQKHGLERFDPGEPGEDGKPQKFDPKIHEATFMTKVEGKENGEIMYT
          	:*****:.**:.*: ******:.* ..**..... *:*: :*****:   .*:.. ::..

MG03214.1 	QQTGFKLNGRVIR~PAKVGVVKNS
NCU01516.1	QQKGFKLNGRVLR~PAQVGVVKNK
FG00305.1 	QQKGFKLNGRVLR~AAKVGVVKNK
AN6248.1  	QSKGFTLNGRVLR0AAKVGVVKND
          	*..**.*****:* .*:******.
```
